# Hepatitis E Outbreak, Dadaab Refugee Camp, Kenya, 2012

**DOI:** 10.3201/eid1906.130275

**Published:** 2013-06

**Authors:** Jamal A. Ahmed, Edna Moturi, Paul Spiegel, Marian Schilperoord, Wagacha Burton, Nailah H. Kassim, Abdinoor Mohamed, Melvin Ochieng, Leonard Nderitu, Carlos Navarro-Colorado, Heather Burke, Susan Cookson, Thomas Handzel, Lilian W. Waiboci, Joel M. Montgomery, Eyasu Teshale, Nina Marano

**Affiliations:** US Centers for Disease Control and Prevention, Nairobi, Kenya (J.A. Ahmed, L.W. Waiboci, J.M. Montgomery, N. Marano);; United Nations High Commissioner for Refugees, Nairobi (E. Moturi, W. Burton);; United Nations High Commissioner for Refugees, Geneva, Switzerland (P. Spiegel, M. Schilperoord);; Kenya Red Cross, Nairobi (N.H. Kassim);; Kenya Medical Research Institute, Nairobi (A. Mohamed, M. Ochieng, L. Nderitu);; US Centers for Disease Control and Prevention, Atlanta, Georgia, USA (C. Navarro-Colorado, H. Burke, S. Cookson, T. Handzel, E. Teshale)

**Keywords:** Hepatitis E virus, refugee camps, Kenya, outbreak, viruses, HEV, acute jaundice syndrome

**To the Editor:** Hepatitis E virus (HEV) is transmitted through the fecal-oral route and is a common cause of viral hepatitis in developing countries. HEV outbreaks have been documented among forcibly displaced persons living in camps in East Africa, but for >10 years, no cases were documented among Somali refugees (*1*,*2*). On August 15, 2012, the US Centers for Disease Control and Prevention (CDC) in Nairobi, Kenya, was notified of a cluster of acute jaundice syndrome (AJS) cases in refugee camps in Dadaab, Kenya. On September 5, a CDC epidemiologist assisted the United Nations High Commissioner for Refugees (UNHCR) and its partners in assessing AJS case-patients in the camp, enhancing surveillance, and improving medical management of case-patients. We present the epidemiologic and laboratory findings for the AJS cases (defined as acute onset of scleral icterus not due to another underlying condition) identified during this outbreak. 

Dadaab refugee camp is located in eastern Kenya near the border with Somalia. It has existed since 1991 and is the largest refugee camp in the world. Dadaab is composed of 5 smaller camps: Dagahaley, Hagadera, Ifo, Ifo II, and Kambioos. As of December 2012, a total of 460,000 refugees, mainly Somalians, were living in the camps; >25% were recent arrivals displaced by the mid-2011 famine in the Horn of Africa (*3*). Overcrowding and poor sanitation have led to outbreaks of enteric diseases, including cholera and shigellosis (*4*); in September 2012, an outbreak of cholera occurred simultaneously with the AJS outbreak.

During July 2–November 30, 2012, a total of 339 AJS cases were reported from the camps and 2 nearby villages: 232 (68.4%) from Ifo II, 57 (16.8%) from Kambioos, 26 (7.7%) from Ifo, 12 (3.5%) from Dagahaley, 10 (3.0%) from Hagadera, and 1 each (0.6%) from the nearby Kenyan villages of Biyamadow and Darkanley. The epidemic curve of the outbreak is shown in the Figure.

**Figure:**
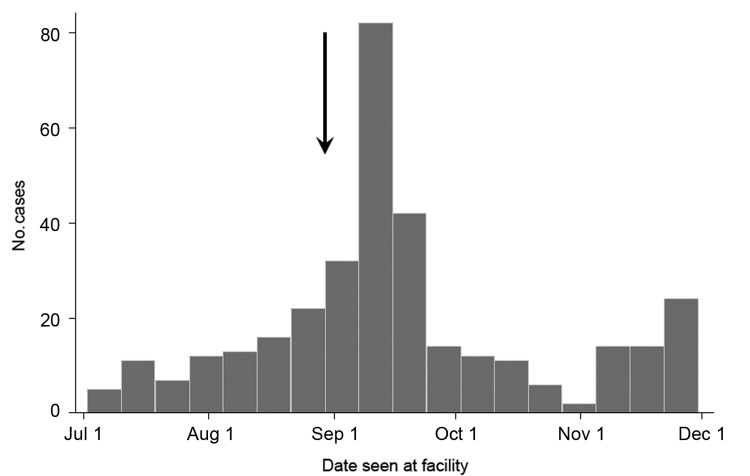
Cases of acute jaundice syndrome, Dadaab, Kenya, July–November 2012. The arrow indicates the point at which outbreak control measures (e.g., construction of new latrines and hygiene messaging) were initiated by health authorities.

Of the 339 AJS case-patients, 184 (54.3%) were female. The overall median age was 23.5 years (range 1 month−91 years). The median age among female and male residents was 24 years and 20 years, respectively. Among the 134 women of reproductive age (15−49 years), 72 (53.7%) reported being pregnant; the median gestational age was 17.4 weeks (range 8.7−35.3 weeks). Death was reported for 10 of the 339 case-patients (case-fatality ratio 2.9%), 9 of whom were postpartum mothers (case-fatality ratio 12.5%) and 1 a 2-year-old child. 

Serum samples were obtained from 170 (50.1%) AJS case-patients for testing at the Kenya Medical Research Institute/CDC laboratories in Nairobi, Kenya. Of the 170 samples, 148 were tested for hepatitis E virus (HEV) IgM by using an ELISA (Diagnostic Systems, Saronno, Italy), and 93 were tested for HEV RNA by using the GeneAmp Gold RNA PCR Reagent Kit (Applied Biosystems, Foster City, CA, USA). Of the 170 samples tested, 131 (77.1%) were positive for HEV IgM, HEV RNA, or both: 120 (81.1%) of 148 tested for HEV IgM and 48 (51.6%) of 93 tested for HEV RNA were positive. In response to the outbreak, UNHCR and partners initiated control measures, including training of health care workers, increasing community awareness, improving hygiene promotion activities, and hastening latrine construction.

The outbreak also affected refugee resettlement to the United States and other countries. At the onset of the outbreak, ≈100 Dadaab refugees per month were scheduled for US resettlement. The incubation period for HEV is 15–60 days (*5*); thus, there was concern that refugees could become ill in transit or within weeks of US resettlement. Acute HEV infection, including progression to fulminant hepatitis, had been reported among travelers returning from regions where the disease is endemic (*6*). As a precaution, the International Organization for Migration and CDC conducted heightened AJS surveillance during pre-departure and arrival health screenings. As of February 2013, no cases of AJS were reported among refugees from Dadaab who resettled in the United States.

Dadaab has faced grave insecurity: aid workers were abducted from the camp in late 2011, and Dadaab has experienced numerous blasts from explosive devices (*7*). Thus, UNHCR and CDC have been limited in their capacity to collect data and conduct a thorough outbreak investigation to identify risk factors. An earlier study in the Shebelle region of Somalia suggested an increased incidence of HEV during the rainy season and elevated risk for infection in villages dependent on river water (*8*). Further evaluation is needed to identify the risk factors for HEV transmission and HEV-associated deaths in this region, including the role of person-to-person transmission. UNHCR and CDC investigations of HEV outbreaks in refugee camps in southern Sudan may provide data to answer these questions.

HEV is believed to have infected humans for centuries (*9*); however, the reemergence of the disease in refugee camps is a major concern because of the difficulty in implementing effective preventive measures under camp conditions. Point-of-care tests will be useful for rapidly detecting outbreaks and could potentially save lives. The progress made in developing effective vaccines is encouraging (*10*). Once available, HEV vaccination should be prioritized in this population, especially for pregnant women.
